# The impacts of digital payment capability on food consumption upgrading in rural households: evidence from China

**DOI:** 10.3389/fnut.2025.1703317

**Published:** 2025-12-16

**Authors:** Chao Zhou, Shiyuan Wang, Lei Zhou, Shenwei Wan

**Affiliations:** 1 Research Center of the Economic and Social Development of Henan East Provincial Joint, Shangqiu Normal University, Shangqiu, China; 2 School of Agricultural Economics and Rural Development, Renmin University of China, Beijing, China

**Keywords:** digital payment capability (DPC), rural households, the upgrading of food consumption pattern (UFCP), ford behavioral model, sustainable development goals (SDGs)

## Abstract

Globally, rural areas in developing countries face food insecurity and malnutrition, threatening both public health and sustainable development. This study examines the impact of digital payment capability (DPC) on the upgrading of food consumption pattern (UFCP) among rural Chinese households. Guided by Sen’s Feasible Capability framework, we construct a multidimensional DPC index comprising information access, liquidity access, digital engagement and external training. We apply this index to 2,078 households from the China Rural Revitalization Survey (CRRS) and adopt the Ford Behavioral Model to analyze both direct effects and mediating pathways. Our main findings are as follows. First, DPC significantly enhances UFCP, with the effect largely mediated by increases in household income and improvements in the health awareness of household heads. Second, the positive association between DPC and UFCP is strongest in eastern and western provinces, in peri-urban areas and in regions with more advanced digital economies. Third, dietary gains from higher DPC are most pronounced in households led by middle-aged, less-educated or less-healthy individuals, and in families with middle to high incomes. These results underscore the potential of digital payment services to drive dietary transformation in rural China. To maximize impact, policymakers should expand digital-payment infrastructure and pair it with targeted income-support and health-literacy programs, thereby fostering more diverse diets and greater well-being in rural populations.

## 1 Introduction

On September 25, 2015, the United Nations Development Summit adopted Transforming Our World: The 2030 Agenda for Sustainable Development. The Agenda covers 17 Sustainable Development Goals (SDGs), of which Goal 3 emphasizes “ensuring healthy lifestyles and promoting well-being for all at all ages” (1). More than 2.8 billion people were unable to afford a healthy diet in 2022, a figure particularly significant in low-income countries, where the proportion of the population unable to a afford healthy diet is as high as 71.5 percent.^1^ This trend not only leads to impaired growth and development in children, but also significantly increases the healthcare costs for poor families, posing a serious threat to socio-economic development. Therefore, improving the nutritional status of families in backward areas is of great significance for enhancing the health and well-being of all mankind.

As the world’s largest developing country, the new situation China is facing is that its disease spectrum has shifted from being mainly characterized by infectious diseases to being mainly characterized by chronic non-communicable diseases, and the trend of younger patients with chronic diseases is obvious (2). In 2018, the top four mortality rates for major diseases among both urban and rural residents in China were attributed to chronic diseases, in descending order as follows: malignant tumors, heart diseases, cerebrovascular diseases, and respiratory diseases. At the same time, there are more pronounced urban–rural differences in the disease structure in China (3). At present, the incidence of anemia and vitamin A deficiency, as well as cardiovascular and cerebrovascular disease mortality and stroke, is significantly higher among rural residents than among urban residents. Unhealthy dietary patterns are the most important factor in the prevalence of chronic diseases and mortality in China (4). The per capita consumption level of high-nutrition foods such as fish, poultry, milk and fruits among rural residents in China remains relatively low, and the food consumption structure of rural households needs to be further optimized.

As the global digital transformation accelerates, digital payment is profoundly changing people’s daily life, especially in the area of food consumption (5). In rural areas, digital payments exert a direct influence on food consumption choices beyond mere transaction facilitation. First, reductions in transaction costs and enhancements in perceived immediacy and convenience directly raise the share of highly convenient and pre-packaged foods in household diets, reflecting a shift in consumption driven by increased purchasing power. Second, the expansion of financial services accompanying digital payments—such as microcredit, installment purchases, and subsidy transfers—directly relaxes budget constraints and broadens households’ feasible food portfolios toward affordable, nutrient-dense options, thereby elevating both income and dietary awareness as drivers of consumption. Third, the integration of digital payments with big data, personalized promotions, and an expanding network of local merchants directly affects information accessibility and promotional exposure, enhancing nutrition-related knowledge and informing healthier choices, which in turn influences consumption patterns. Finally, widespread digital payments may alter risk perceptions and access to food-safety information, increasing trust in local high-quality or organic foods and thereby shifting consumption patterns.

Rural China has a huge population, and its food consumption pattern has a profound impact on global sustainable development. Therefore, it is of great practical significance for this paper to conduct in-depth research on the impact of digital payment capability (DPC) on the upgrading of food consumption pattern (UFCP) in rural households by using large-scale rural survey data in China. It can not only provide a scientific basis for the formulation of poverty reduction policies, but also contribute to improving global public health. Through this study, we expect to provide new perspectives and strategies for the transformation of rural development and food consumption pattern worldwide. To ascertain whether DPC exerts a direct influence on UFCP in rural households, this study conducts a quantitative assessment of the levels of UFCP and DPC among 2,078 rural households covering 10 provinces of China in 2020. Subsequently, an in-depth analysis delves into the ramifications of DPC on UFCP in rural households, scrutinizing its impact mechanisms. This investigation further delineates the varied impacts of DPC on UFCP, examining disparities attributable to geographic location, individual characteristics, and household income.

## 2 Literature review

FAO indicated a substantial rise in per capita meat supply from roughly 4 kg per year in 1962 to about 70 kg per year in 2022, reflecting persistent diversification of the diet. Currently, Chinese residents consume fewer grain-based staples than a decade ago, while intakes of complementary foods such as meat, eggs, dairy, fruits, and vegetables have generally risen (6–8). These foods are characterized by diversity, convenience, nutrition, safety and health. The study finds that UFCP has a significant impact on the demand for arable land, the agricultural production structure, agricultural carbon emissions, water footprint, and nitrogen footprint (9–14). Academic research on rural residents’ food consumption has mainly focused on its determinants. Income, expenditure, and price are the primary determinants affecting rural food consumption. Many scholars have estimated the price, income, and expenditure elasticities of demand for food consumption using the demand system model (15, 16). Macroeconomic factors such as agricultural mechanization, urbanization, level of marketization, aging, and epidemic shocks are also key drivers of changes in food consumption patterns among rural residents (17). In addition, individual characteristics and subjective cognitive factors such as migration status, education level, income expectations, and nutritional attitudes of decision makers have a significant impact on UFCP among rural residents (18, 19).

The rapid advancement of digital technology and the digital economy is driving broad digital transformations in production and daily life, a trend that has intensified in rural contexts as digital infrastructure expands (20–23). Across recent work, scholars have documented how digitalization reshapes rural consumption patterns through multiple channels. First, a strand of literature emphasizes determinants of food consumption rooted in broader structural factors such as Internet access, network infrastructure, and e-commerce platforms, highlighting how digital inclusion can raise overall consumption levels and diversify rural diets (24). Second, there is a growing body of work on digital finance as a driver of rural consumption, identifying mechanisms including rural industrial development, new business forms, and enhanced financial accessibility that collectively stimulate demand (25, 26). A third cluster finds that digital payments may exert a direct effect on consumption by easing liquidity constraints and enabling consumer credit, thereby elevating household spending on food and related goods (27, 28). Fourth, studies addressing behavioral dimensions suggest threshold effects and potential distributive consequences of digitalization, underscoring concerns about the digital divide, shifts in consumption choices driven by technology, and implications for food security. Across these strands, several results converge on the notion that digital finance and payment ecosystems can enhance rural consumption, yet important gaps remain regarding the relative importance of direct versus indirect channels, regional heterogeneity, and the integrative role of nutritional quality in the upgrading of rural diets. This study contributes by articulating a multidimensional DPC framework and by linking DPC to UFCP through income and health-awareness channels, thereby addressing the literature gaps on the mechanisms and context-dependence in rural China. This study advances the literature on DPC and the upgrading of UFCP in three substantive ways. First, drawing on Sen’s Feasible Capability framework, we operationalize a multidimensional DPC index that encompasses information access, liquidity access, digital engagement, and external training, and we propose a comprehensive UFCP indicator that captures both consumption shares and nutrient-intensity dimensions. This approach not only refines measurement of UFCP but also illuminates the structural components through which DPC may shape dietary outcomes. Second, to our knowledge, this work provides the first empirical examination of the link between DPC and UFCP in rural households, thereby enriching the discourse on digital finance as a driver of dietary change and offering empirical support for the broader welfare implications of digital technologies in underserved contexts. Third, situating the analysis within the Fogg Behavior Model, we identify the channels through which DPC operates to influence UFCP, yielding new theoretical and practical insights for policies aimed at expanding digital payments and improving dietary quality in rural populations. Taken together, these contributions provide a rigorous empirical and conceptual foundation for policy design seeking to promote digital payment adoption and enhance nutritional outcomes in less developed regions.

## 3 Theoretical models and research hypotheses

### 3.1 Direct impact of DPC on UFCP

Compared with traditional means of payment, digital payment is characterized by security, speed and the ability to transcend time and space constraints, which can reduce transaction costs. Digital payment alleviates short-term liquidity constraints through consumer credit, dulls the perception of payment pain, and increases price tolerance, thereby promoting consumption among rural households (25). DPC enables rural residents to fully enjoy the benefits of digital payment and enhances their access to and willingness to purchase food with high nutritional value, thus contributing to the upgrading of food consumption pattern (29). Drawing from the preceding theoretical analysis, this paper formulates the following hypothesis.

*H1*: DPC significantly promotes UFCP in rural households.

### 3.2 Indirect impact of DPC on UFCP

Fogg (30) proposed a new model for understanding consumer behavior decisions, namely the Fogg Behavior Model (FBM). According to this model, the implementation of consumption decisions relies on three elements: Motivation, Ability, and Trigger. Only when all three elements converge simultaneously can a purchase behavior be triggered, that is, B = MAT. Based on the FBM model, this paper holds that health awareness serves as the motivation for food consumption decisions, while household income constitutes the capacity constraint on such decisions. By effectively leveraging the convenience of digital payment and the lower psychological loss associated with it, DPC functions perfectly as a trigger.

DPC can increase household income and improve rural residents’ accessibility to highly nutritious food. Firstly, DPC can boost the business income of rural households. The improvement of DPC facilitates farmers in expanding online sales channels, directly connecting with consumers, and thus increasing profit margins. At the same time, farmers can use digital platforms to publicize their products, build a distinctive brand, and achieve brand premiums (31, 32). Secondly, DPC can increase the wage income of rural residents. The acquisition of DPC can provide access to more vocational training resources and improve their skill quality and employability (33). Moreover, rural residents have access to more information on non-farm employment at a lower cost, which in turn creates conditions for increased wage income (34). Thirdly, DPC can increase the property income of rural residents. By enhancing their DPC, rural residents can access more high-quality financial products and optimize their asset allocation, thereby enhancing their property income. Empirical studies also show that improved digital skills have an income-generating effect, significantly contributing to business income, wage income, and property income (35). Income is an important basis for influencing rural residents’ consumption. According to Bennett’s law, higher incomes significantly increase the proportion of highly nutritious food in the food consumption structure, thereby upgrading food consumption (36). Based on this, the second hypothesis of this paper is proposed.

*H2*: The improvement of DPC significantly promotes UFCP in rural households by boosting their income.

DPC can enhance the health awareness of household heads and their willingness to consume highly nutritious food. One of the important ways the Internet affects the consumption of rural residents is by changing their consumption concepts (37). Firstly, the improvement of DPC enables rural residents to access a wealth of healthcare information online, expand their channels for obtaining dietary and nutritional information, which is conducive to fostering their awareness of healthy diet (38). Secondly, Internet platforms can create personalized consumer profiles through algorithms, deliver relevant products and services to consumers more accurately based on their daily searches for healthcare information, thereby enhancing their willingness to consume nutritionally rich foods (39). Finally, digital platform realizes the direct matching of supply and demand. Empirical studies have also shown that digital payment can promote household preventive health investments through the health consumption awareness of family members. The improvement of DPC can lead rural residents to develop the intention to purchase healthy and nutritious food within the rich application scenarios of the internet, thereby promoting UFCP in rural households. Based on this, the third hypothesis of this paper is proposed.

*H3*: The improvement of DPC promotes UFCP in rural households by enhancing the health awareness of rural residents.

This paper also constructs a microeconomic model to demonstrate how DPC affects UFCP in rural households. Suppose that the utility function of consumers relies on the consumption of both normal food and food with high nutritional value. The following variables and functions are defined in this paper:

$U(C_{t},C_{u},H)$ : Consumer’s utility function, where $C_{t}$ represents the consumption of normal food, $C_{u}$ represents the consumption of food with high nutritional value, and H represents the consumer’s level of health awareness.

I : Consumer’s initial income.

$P_{t}$ : The average price of normal food.

$P_{u}$ : The average price of food with high nutritional value.

D: Digital payment capability.

Assume that the utility function is of Cobb–Douglas form and consider that consumers’ health awareness influences their preference for food with high nutritional value (Equations 1–6):


$$U(C_{t},C_{u})=C_{t}^{\alpha }C_{u}^{\beta }H^{\varphi }(\alpha +\beta =1)$$ (1)

The budget constraint:


$$P_{t}C_{t}+P_{u}C_{u}=I\prime$$ (2)

The effect of the DPC on disposable income and health awareness is assumed to be linear.


I′=I+θD(θ>0) (3)

$$H=H_{0}+\mathrm{\gamma D}(\gamma >0)$$ (4)

Constructing a Lagrangian function to determine the consumers’ optimal consumption choices.


$$L(C_{t},C_{u},\lambda )=C_{t}^{\alpha }C_{u}^{\beta }{\left(H_{0}+\mathrm{\gamma D}\right)}^{\varphi }+\lambda (I\prime -P_{t}C_{t}-P_{u}C_{u})$$ (5)

Determine the optimal consumption of $C_{u}$ by taking the first-order derivative of $C_{t}$ , $C_{u}$ , and λ and setting them to zero.


$$C_{u}=\frac{\mathrm{\alpha I}\prime }{P_{u}}=\frac{\alpha (I+\mathrm{\theta D})}{P_{u}}$$ (6)

With the increase of DPC, consumers tend to increase the consumption of food with high nutritional value, thus promoting UFCP in rural households.

**Figure 1:**
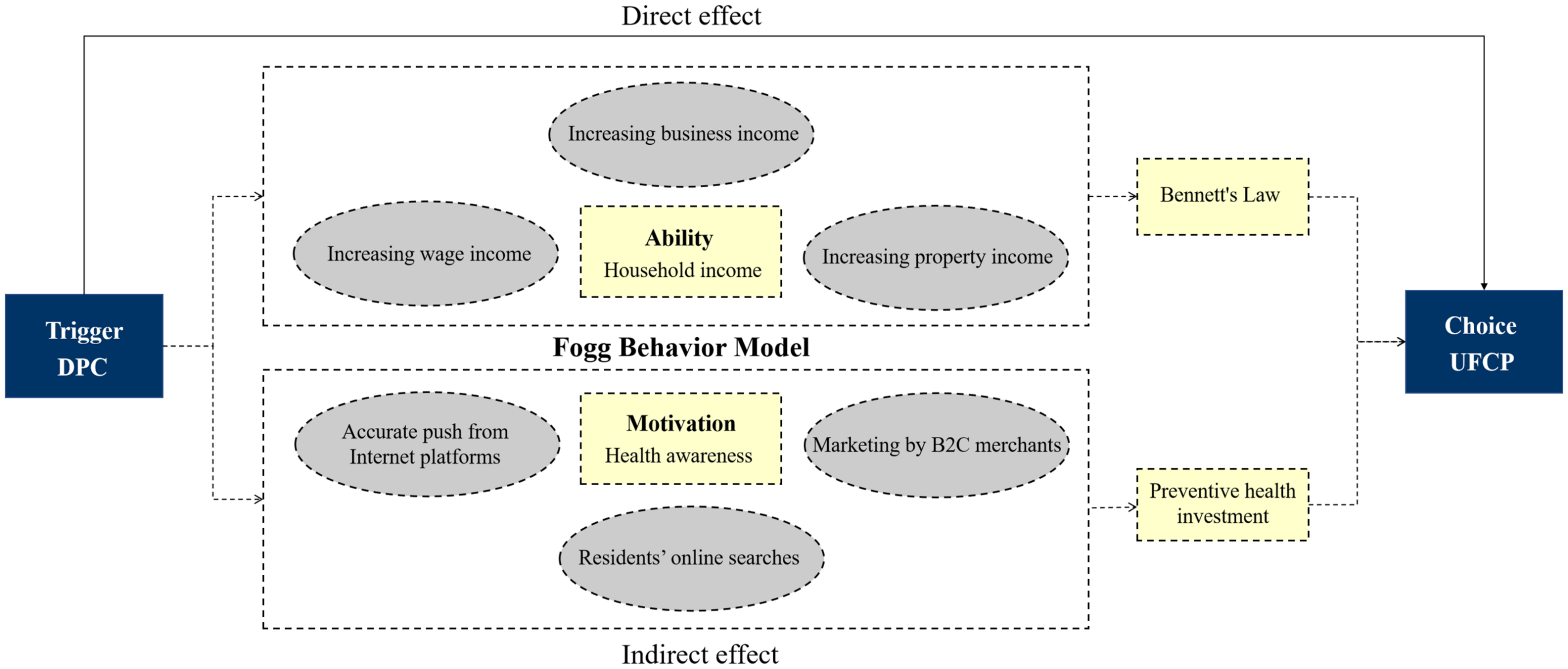
Theoretical mechanism diagram.

A concise synthesis of the theoretical mechanisms is as follows: DCP enhances disposable income and health awareness of rural residents, which jointly increase demand for nutritionally rich foods, yielding both direct and indirect effects on UFCP, The details of the theoretical framework are depicted in Figure 1.

## 4 Research design

### 4.1 Data source

The data involved in this study comes from the China Rural Revitalization Survey (CRRS) conducted by Rural Development Institute, Chinese Academy of Social Sciences. The structured questionnaire survey was conducted from August to September 2020, covering many aspects such as agricultural production, rural development, farmers’ lives and social welfare. The project used a multistage sampling approach for data collection. The CRRS employed a multi-stage stratified random sampling method to select survey samples, covering 10 provinces, 50 counties (cities), 150 towns, and 300 villages across the country. First, considering factors such as economic development level, regional location, and agricultural development, 1/3 of the provinces from the eastern, central, western, and northeastern regions were randomly selected, namely Zhejiang, Shandong, Guangdong, Anhui, Henan, Guizhou, Sichuan, Shaanxi, Ningxia, and Heilongjiang. Then, all counties in each province were sorted according to per capita GDP and divided into five groups: high, upper-middle, middle, lower-middle, and low levels. One county was randomly selected from each group, resulting in a total of 50 sample counties. Next, using the same method, three towns (high, middle, and low economic levels) were selected from each county, and two villages (one with better economic development and one with poorer economic development) were selected from each town. Finally, based on the household registration list provided by the village committee, surveyor’s first selected households residing at home, sorted them, and then used an equal-distance random sampling method to select 14 households, with two households as backups. This study obtained 2,078 rural household samples after deleting samples with missing values and outliers in key variables.

### 4.2 Selection of variables

#### 4.2.1 Dependent variable: upgrading of food consumption pattern (UFCP)

Since 2000, the upgrading of rural Chinese diets is defined here as the shift toward higher-protein, nutrient-dense foods within total food consumption, operationalized as the share of meat, eggs, milk, and fresh vegetables.^2^ This definition reflects the long-run national and regional dietary transitions toward more diverse and richer protein sources, consistent with the literature on dietary upgrading (40). We justify this choice because these items capture the dietary quality dimension most closely linked to nutrition security and the functional use of digital payment to expand access to diverse foods. As a robustness check, we also estimate an alternative specification (UFCP1) using the expenditure shares of meat, eggs, milk, and fresh vegetables in total food expenditure.

#### 4.2.2 Independent variable: digital payment capability (DPC)

Sen’s “Feasible Capability” theory emphasizes the actual freedom of individuals. It is not merely the possession of resources, but the capacity to convert these resources into practical actions and states (41). This article defines DPC as the ability of an individual or household to use digital technology to participate in digital application scenarios and enhance their welfare based on certain subjective and objective conditions, including information access, liquidity access, digital engagement, and external training. First, DPC fundamentally depends on access to appropriate information infrastructure. Accordingly, household Internet devices were coded as follows: smartphones were assigned a value of 2, non-smartphones a value of 1, and devices without Internet access a value of 0. Second, financial resources are essential for DPC. For the questions “What is the amount of money in your WeChat wallet balance, Alipay balance, and cash?” and “What is the total overdraft limit available on your credit card, Ant Credit Pay, JD Credit, and similar services?,” responses indicating a positive amount were coded as 1, and zero or a negative amount as 0. Third, DPC is also reflected in participation in information-driven production and consumption activities. Thus, for the survey questions “Does your household sell products online?” and “Have you ever paid fees for mobile application services?,” affirmative responses were coded as 1 and negative responses as 0. Finally, DPC may be enhanced through external training; therefore, respondents who answered “yes” to the question “Have you received any training in computer use or mobile internet?” were assigned a value of 1, while “no” responses were assigned 0. The composite digital payment capability index was constructed by summing these component scores.

#### 4.2.3 Control variables

Drawing on the research of Bai et al. (42), this paper selects the individual characteristics of the household head, family characteristics of the respondents and village characteristics as control variables. Individual characteristics mainly include gender, age, marital status, educational level, health condition, and job status. Family characteristics mainly include family size, proportion of the elderly population, proportion of children, annual family income, and family housing assets, since larger households and younger-age structures influence economies of scale, dietary needs, and spending priorities, which could otherwise confound estimates of the direct and indirect effects of digital payment capability on food consumption. Village characteristics mainly include the distance from the village committee to the county center and the per capita disposable income of the village.

### 4.3 Descriptive statistics

The definitions and descriptive statistics of the main variables are presented in Table 1. The mean values of UFCP and DPC among rural households in the sample data are 0.307 and 2.292, respectively. As indicated in Table 2, the mean values of UFCP and DPC for male-headed rural households are 0.294 and 2.279, respectively, while for female-headed households, the values are 0.350 and 2.234, respectively. This indicates that the degree of UFCP is higher in female-headed households than in male-headed ones. Gender differences in UFCP and DPC likely reflect unequal access to information and nutrition knowledge plus differing intra-household dynamics. However, the DPC of male-headed households is slightly higher than that of female-headed households. As reported in Table 2, the mean values of UFCP and DPC for married-headed rural households are 0.308 and 2.295, respectively, while for unmarried-headed households, the values are 0.293 and 2.239, respectively. This indicates that there is a greater difference in UFCP between married-headed and unmarried-headed households, and that DPC of married-headed households is slightly higher than that of unmarried-headed households. There are large differences in the degree of UFCP and DPC among rural households in different regions of China. Rural households in the central and western regions have notably lower UFCP and DPC compared to those in the eastern region (See Table 2).

**Table 1:** Definition and descriptive statistics of the main variables.

Variable label	Definition	Mean (S.D.)
Dependent variables		
UFCP	Proportion of meat, eggs, milk, and fresh vegetable purchases in total food consumption (%)	0.307 (0.223)
Independent variables		
DPC	A comprehensive indicator, as referenced above	2.292 (0.914)
Control variables		
Gender	Male = 1; Female = 0	0.767 (0.423)
Age	Age of the household head (years)	53.590 (10.980)
Age2	Age squared /100	29.920 (11.720)
Marriage	Married = 1; Unmarried = 0	0.932 (0.252)
Edu	No Schooling = 1; Primary School = 2; Junior High School = 3; Senior High School = 4; Technical Secondary School = 5; Vocational High School = 6; College = 7; Undergraduate = 8; Postgraduate = 9	2.897 (1.167)
Village_cadre	Is a village cadre = 1; Not a village cadre = 0	0.224 (0.417)
Health	Compared with peers: Very bad = 1, Poor = 2, Average = 3, Good = 4, Very good = 5	3.631 (0.981)
Familysize	Number of household members	4.008 (1.477)
Child_pro	Proportion of household members under 15 years old	0.131 (0.167)
Elderly_pro	Proportion of household members over 60 years old	0.219 (0.313)
lnincome	Sum of net business income, wage income, property income, and net transfer income (Yuan), take logarithm	10.710 (1.134)
Houses	Number of residential plots	1.179 (0.444)
County_distance	Distance from the village committee to the county government (100 km)	0.229 (0.165)
lnvillage_income	Disposable income of the village per capita (Yuan), take logarithm	9.404 (0.708)

**Table 2:** Heterogeneous results of the dependent and independent variables.

Categories		Variables	*N*	Mean	SD	Min	Max
Region	Eastern	UFCP	506	0.394	0.230	0	1
		DPC	506	2.314	1.029	0	6
	Central	UFCP	615	0.271	0.209	0	0.993
		DPC	615	2.237	0.887	0	6
	Western	UFCP	957	0.284	0.216	0	1
		DPC	957	2.251	0.864	0	6
Gender	Male	UFCP	1,593	0.294	0.219	0	1
		DPC	1,593	2.279	0.915	0	6
	Female	UFCP	485	0.350	0.232	0	1
		DPC	485	2.234	0.911	0	6
Marital status	Married	UFCP	1,936	0.308	0.223	0	1
		DPC	1,936	2.295	0.899	0	6
	Unmarried	UFCP	142	0.293	0.217	0	0.895
		DPC	142	2.239	1.098	0	6

### 4.4 Model specifications

#### 4.4.1 Benchmark regression model

To test the impact of DPC on UFCP in rural households, a basic econometric model is established:


$$\mathrm{UFC}P_{i}=\alpha +\mathrm{\beta DP}C_{i}+\text{γcontrol}s_{i}+\varepsilon_{i}$$ (7)

In Equation 7, $\mathrm{UFC}P_{i}$ represents UFCP in rural household i; $\mathrm{DP}C_{i}$ represents the DPC of the head in rural household i; $\text{control}s_{i}$ represent the Individual characteristic variables, family characteristic variables, and village characteristic variables; $\varepsilon_{i}$ is the error term.

#### 4.4.2 Mediation effect model

The three-step mediation model examines pathways through household income and health awareness (43):


$$M_{i}=\alpha +\mathrm{\beta DP}C_{i}+\text{γcontrol}s_{i}+\varepsilon_{i}$$ (8)

$$\mathrm{UFC}P_{i}=\alpha +\mathrm{\beta DP}C_{i}+M_{i}+\text{γcontrol}s_{i}+\varepsilon_{i}$$ (9)

In Equations 8, 9, M denotes the mediating variables, which are household income (H_income) and health awareness (H_awareness) respectively, and the other letters have the same meaning as in Equation 7. Health awareness is measured by the question “Do you consciously seek out health or wellness knowledge?,” with affirmative responses coded as 1 and negative responses coded as 0. This measure reflects respondents’ active engagement in acquiring health-related information. Household income is represented by annual per capita family income, which accounts for total household income divided by the number of family members, providing a standardized indicator of economic resources.

Together, the benchmark and moderating models formalize how DCP translates into dietary upgrading through both direct channels and mediation via income and health awareness. The specification aligns with established mediation frameworks and is complemented by robustness checks to ensure conclusions are robust to regional heterogeneity and potential confounders.

## 5 Results

### 5.1 Benchmark regression

This paper uses the stepwise regression method to perform benchmark regression (Table 3). Model (1) does not consider control variables; Model (2)–Model (4) gradually add control variables for the individual characteristics of the household head, family characteristics, and village characteristics. The results reveal a statistically significant positive coefficient for DPC at the 1% level, indicating that the improvement of DPC notably promotes UFCP in rural households. These outcomes provide robust support for H1.

**Table 3:** Results of the benchmark regression.

Variables	Model (1)	Model (2)	Model (3)	Model (4)
	UFCP	UFCP	UFCP	UFCP
DPC	0.055*** (10.45)	0.037*** (5.95)	0.031*** (4.93)	0.032*** (5.06)
Gender		−0.058*** (−5.10)	−0.059*** (−5.24)	−0.051*** (−4.55)
Age		−0.001 (−0.47)	0.001 (0.19)	−0.003 (−0.80)
Age2		0.001 (0.37)	−0.000 (−0.14)	0.003 (0.78)
Marriage		0.017 (0.87)	0.012 (0.64)	0.009 (0.48)
Edu		0.018*** (3.97)	0.017*** (3.59)	0.012** (2.58)
Health		0.019*** (3.84)	0.016*** (3.28)	0.016*** (3.35)
Village_cadre		0.019 (1.61)	0.018 (1.54)	0.020* (1.75)
Familysize			−0.008** (−2.10)	−0.008** (−2.07)
Child_pro			0.123*** (3.58)	0.116*** (3.42)
Elderly_pro			0.011 (0.49)	−0.000 (−0.01)
lnincome			0.021*** (4.47)	0.019*** (4.05)
Houses			0.029*** (2.75)	0.029*** (2.72)
lnvillage_income				0.015** (2.16)
County_distance				−0.192*** (−6.56)
Constant	0.182*** (14.14)	0.172* (1.89)	−0.105 (−0.98)	−0.074 (−0.61)
*N*	2,078	2,078	2,078	2,078
*R* ^2^	0.050	0.079	0.097	0.121

The *t*-values are in parentheses; *, **, and *** indicate significance at the 10, 5, and 1% levels, respectively.*p < 0.1, **p < 0.05, ***p < 0.01.

### 5.2 Endogeneity treatment

The benchmark regression model has potential endogeneity issues such as reverse causality and omitted variable bias (44). With the upgrading of food consumption pattern, rural residents gradually develop the habit of consuming high-quality foods. Affected by the ratchet effect, rural residents are more willing to improve their digital payment capability to meet their quality of life needs (16). To address potential endogeneity concerns that could bias the results, this study employs an instrumental variable (IV) approach. This paper selects the average DPC of all households in the same county other than this household, as an instrumental variable for endogenous treatment (45). This instrumental variable is correlated with a certain household’ DPC. At the same time, the average DPC of other households in the same county does not directly affect the consumption pattern of a randomly selected household, thus satisfying the exogeneity assumption of IV. The results from the 2SLS regression model are presented in Table 4. The *F* value in the first stage is significantly greater than 10, indicating that the instrumental variable is not weak. The estimation results from the second stage show that the regression coefficient of DPC remains significantly positive, indicating that the basic conclusion of this paper is robust even after controlling for potential endogeneity issues.

**Table 4:** Results of endogeneity testing.

Variables	First-stage regressions	IV (2SLS) estimation
	DPC	UFCP
IV	0.374*** (6.71)	
DPC		0.084* (1.94)
Constant	1.446*** (3.39)	−0.179 (−1.20)
Controls	Yes	Yes
*N*	2,078	2,078
*R* ^2^	0.368	0.091
*F*-value	80.18	

The *t*-values are in parentheses; *, **, and *** indicate significance at the 10, 5, and 1% levels, respectively.*p < 0.1, **p < 0.05, ***p < 0.01.

### 5.3 Robustness testing

#### 5.3.1 Replacing the dependent variable

This paper replaced the dependent variable UFCP with the upgrading of food consumption pattern measured in monetary terms (UFCP1). The regression results are shown in Model (5) of Table 5, where the coefficient of DPC is significantly positive. This finding strongly supports the conclusion that DPC effectively promotes UFCP in rural households, providing robust evidence for the basic conclusion of this study.

**Table 5:** Results of robustness tests.

Variables	Model (5)	Model (6)	Model (7)	Model (8)	Model (9)
	UFCP1	UFCP	UFCP	UFCP	UFCP
DPC	0.016** (2.30)		0.032*** (5.06)	0.030*** (4.83)	0.027*** (4.51)
DPC1		0.081*** (4.75)			
Constant	−0.048 (−0.35)	−0.142 (−1.15)	−0.074 (−0.61)	−0.239* (−1.68)	−0.264 (−1.49)
*N*	2,078	2,078	2,078	2,078	2,078
*R* ^2^	0.122	0.120	0.121	0.125	0.125

The *t*-values are in parentheses; *, **, and *** indicate significance at the 10, 5, and 1% levels, respectively.*p < 0.1, **p < 0.05, ***p < 0.01.

#### 5.3.2 Replacing the independent variable

This paper redefines the independent variable (DPC1), where DPC1 values ranging from 0 to 3 represent weak digital payment capability, while values ranging from 4 to 6 signify strong digital payment capability. The results in Model (5) of Table 5 show that digital payment capability maintains a pronounced positive influence on UFCP at the 1%significance threshold after changing the measurement method.

#### 5.3.3 Replacing the regression model

This paper employs Tobit model to account for potential distributional truncation. As shown in Model (7) of Table 5, the regression results indicate that the coefficient of DPC is significantly positive at the 1% level, corroborating the results obtained from the primary regression analyses conducted in this study.

#### 5.3.4 Outlier treatment

In order to eliminate the impact of extreme values, this paper performed winsorization by 1 and 5% on all continuous variables. Model (8) and Model (9) of Table 5 show that the coefficient of DPC remains significantly positive. This finding suggests that, even after dealing with the outliers, DPC continues to exert a positive effect on UFCP in rural households.

### 5.4 Mechanism validation

The estimation results of the benchmark regression indicate that DPC has a significant promoting effect on UFCP in rural households. To further analyze the underlying mechanisms of this effect, this section integrates the theoretical explanatory framework developed in the previous section with the established research framework to examine two key dimensions: household income and health awareness. Mediation analyses using the three-step approach show significant positive coefficients for DPC on H_income and H_awareness, and for these mediating variables on UFCP, with the significance of DPC on UFCP remaining robust after inclusion of mediating variables (see Table 6). Household income and health awareness play a partial mediating role in the process of DPC affecting UFCP and all pass the Sobel test. In summary, the improvement of DPC has contributed positively to the promotion in UFCP by raising the household income and the health awareness of the household head. H2 and H3 are verified.

**Table 6:** Estimated results of impact mechanism.

Variables	Model (10)	Model (11)	Model (12)	Model (13)	Model (14)	Model (15)
	UFCP	H_income	UFCP	UFCP	H_awareness	UFCP
DPC	0.036*** (5.83)	0.238*** (8.18)	0.032*** (5.03)	0.032*** (5.06)	0.075*** (5.25)	0.030*** (4.75)
H_income			0.019*** (4.13)			
H_awareness						0.024** (2.50)
Constant	0.043 (0.37)	6.123*** (11.06)	−0.075 (−0.62)	−0.074 (−0.61)	−0.447 (−1.62)	−0.063 (−0.52)
Controls	Yes	Yes	Yes	Yes	Yes	Yes
Observations	2,078	2,078	2,078	2,078	2,078	2,078
R-squared	0.114	0.144	0.121	0.121	0.086	0.124
Sobel test		3.684*** (0.000)			2.257** (0.024)	

The *t*-values are in parentheses; *, **, and *** indicate significance at the 10, 5, and 1% levels, respectively.*p < 0.1, **p < 0.05, ***p < 0.01 (Since there may be collinearity between annual household income and average annual household income, the annual household income variable was removed during the mechanism test).

### 5.5 Heterogeneity analysis

#### 5.5.1 Classification by geographical location

There are significant differences between the eastern, central, and western regions of China, influenced by geographical environment, history and culture, economic structure, national strategy, and other factors (46). Suburban and non-suburban areas are affected by factors such as traffic conditions, information conditions, and market conditions, resulting in certain differences. Therefore, this article conducts a heterogeneity test on the eastern, central, and western regions and whether the village is suburban. The results show that in the eastern and western regions, DPC has a significant promoting effect on UFCP in rural households, while in the central region, DPC has no significant impact on UFCP (see Table 7). This may be because the eastern region usually has a more developed economy, complete technological infrastructure, high digital payment penetration and residents’ ability to accept and apply new technologies (47). Therefore, the upgrading of food consumption pattern in this region is more common. The western region has also made significant progress in promoting the digital economy and improving infrastructure in recent years. Coupled with the government’s supportive policies, this has accelerated the popularity of digital payment tools and the development of the digital industry. In addition, local residents in the western region tend to have a higher proportion of meat and dairy products in their diets, and as digital payment capability improves, their consumption of meat, eggs, and milk has further increased (48). DPC does not have a significant impact on UFCP among rural households in the central region. This may be due to the fact that the sample provinces in this region are biased towards traditional economies. Interestingly, the improvement of DPC has a greater impact on UFCP among rural households in suburban areas. This may be because these households are closer to the market, have better information and transportation conditions, and have easier access to technical training.

**Table 7:** Results of regional heterogeneity.

Variables	Model (16)	Model (17)	Model (18)	Model (19)	Model (20)	Model (21)	Model (22)
	East	Middle	West	Suburb	Non-suburb	High-digit	Low-digit
	UFCP	UFCP	UFCP	UFCP	UFCP	UFCP	UFCP
DPC	0.035***	0.013	0.041***	0.035**	0.030***	0.038***	0.026***
	(2.63)	(1.23)	(4.49)	(2.26)	(4.37)	(4.31)	(2.88)
Controls	Yes	Yes	Yes	Yes	Yes	Yes	Yes
*N*	506	615	957	398	1,680	1,006	1,072
*R* ^2^	0.084	0.123	0.181	0.105	0.134	0.123	0.146

The *t*-values are in parentheses; *, **, and *** indicate significance at the 10, 5, and 1% levels, respectively.*p < 0.1, **p < 0.05, ***p < 0.01.

In addition, the impact of DPC on UFCP among rural households may also vary depending on the development of the regional digital economy. Based on the China Academy of Information and Communications Technology^3^, this paper divides the sample provinces into areas of high digital economy development and areas of low digital economy development. The results show that the improvement of DPC has a greater role in promoting UFCP among rural households in areas with high digital economy development levels. This may be because the digital infrastructure and digital industries in regions with high digital economy development levels are relatively advanced (49).

#### 5.5.2 Classification by individual characteristics

Because rural residents of different ages, educational levels, and health status face varying financial burdens, the impact of DPC on UFCP in their households may be heterogeneous (50). This paper classifies rural residents’ education levels into low educational level (primary school and below), medium educational level (junior high school), and high educational level (senior high school and above), and removes edu variable from the regression model. Additionally, rural residents are divided into three groups: young (ages 18–40), middle-aged (ages 41–65), and elderly (ages 66 and above), with age and age_2 removed from the model. At the same time, this study divides the samples into two groups according to health status: better health status (average, good and very good) and worse health status (poor and very bad). Accordingly, the health variable is removed from the model.

As presented in Table 8, the results of Model (23), Model (24) and Model (25) show that DPC can better promote UFCP in rural households with household heads of both low and high educational levels. This may be because digital payment is more convenient and reduces the risk of using cash. Rural residents with low educational level can purchase diverse, nutritious, and healthy foods with through the Internet more easily. In contrast, rural residents with high educational level exhibit higher technology acceptance, stronger digital literacy, and a greater willingness to buy high-quality food. As shown in Model (26), Model (27) and Model (28) of Table 8, DPC can significantly promote UFCP in rural households with middle-aged household heads, but have no significant impact on UFCP in rural households with young and elderly household heads. This may be because middle-aged rural residents have higher requirements for the quality and nutritional value of food and have the financial capacity to use digital payments to improve their diet. Young rural residents have poor economic conditions, better health, and a weaker awareness of a healthy diet (51). Although elderly rural residents have a strong need for health preservation, their long-term consumption habits lead them to prefer cash payments and limit their acceptance of digital payment technology. As seen in Model (29) and Model (30) of Table 8, DPC plays a greater role in promoting UFCP among rural households with less healthy household heads. This is likely due to the fact that rural residents with poorer health generally have a stronger awareness of health care.

**Table 8:** Results of group heterogeneity.

Variables	Model (23)	Model (24)	Model (25)	Model (26)	Model (27)	Model (28)	Model (29)	Model (30)
	Low_edu	Mid_edu	High_edu	Youth	Mid_aged	Elderly	Better	Worse
	UFCP	UFCP	UFCP	UFCP	UFCP	UFCP	UFCP	UFCP
DPC	0.049*** (4.17)	0.021** (2.39)	0.035** (2.53)	0.016 (1.03)	0.041*** (5.59)	0.016 (0.86)	0.032*** (4.98)	0.073*** (4.05)
Controls	Yes	Yes	Yes	Yes	Yes	Yes	Yes	Yes
*N*	701	1,015	362	240	1,529	309	1,818	260
*R* ^2^	0.096	0.106	0.115	0.152	0.122	0.095	0.104	0.208

The *t*-values are in parentheses; *, **, and *** indicate significance at the 10, 5, and 1% levels, respectively.*p < 0.1, **p < 0.05, ***p < 0.01.

#### 5.5.3 Classification by household income

Income is a basic factor affecting food consumption (52). The impact of the improvement of DPC on UFCP in rural households with different income levels may be heterogeneous. This paper divides the household samples into five categories according to the annual income of households: lowest-income group (0%–20%), lower-income group (20%–40%), middle-income group (40%–60%), higher-income group (60%–80%), and highest-income group (80%–100%). The relevant regression models exclude lnincome variable from the model. The study has found that the impact of DPC on the upgrading of UFCP in the lowest-income group is not significant, and the positive impact of DPC on UFCP significantly enhances from the lower-income group to the high-income group (see Table 9). This may be because households in the lowest-income group are more focused on meeting their basic living needs. Even if they have a certain ability to utilize digital payment, they may prioritize the cost of food over its quality or variety, which restricts the potential for UFCP (53, 54). Households in the other groups have more disposable income to consider the quality and nutrition of food. Simultaneously, they also have better internet access and richer digital skills, enabling them to frequently access a broader range of product information (55).

**Table 9:** Results of income heterogeneity.

Variables	Model (31)	Model (32)	Model (33)	Model (34)	Model (35)
	Lowest (0%–20%)	Lower (20%–40%)	Middle (40%–60%)	Higher (60%–80%)	Highest (80%–100%)
	UFCP	UFCP	UFCP	UFCP	UFCP
DPC	0.011 (0.80)	0.027** (1.97)	0.031** (2.24)	0.051*** (3.68)	0.041*** (3.17)
Controls	Yes	Yes	Yes	Yes	Yes
*N*	416	416	415	416	415
*R* ^2^	0.119	0.084	0.117	0.100	0.133

The *t*-values are in parentheses; *, **, and *** indicate significance at the 10, 5, and 1% levels, respectively.*p < 0.1, **p < 0.05, ***p < 0.01.

## 6 Conclusion

This study investigates the impact of DPC on UFCP in rural households from a micro-level perspective. The results are multifaceted, drawing from both theoretical and empirical research. First, following Sen’s Feasible Capability framework, this paper comprehensively assesses the level of digital payment capability from four aspects: information access, liquidity access, digital engagement, and external training. Second, the baseline regression analysis indicates that the improvement of DPC significantly promotes UFCP in rural households. Robustness tests further support this conclusion. Third, heterogeneity analysis reveals that this effect is particularly pronounced in the eastern and western regions of China, suburbs, and areas with a higher digital economy development level. Importantly, the benefit is concentrated among rural residents who are middle-aged, have a lower level of education, or are in poorer health. Moreover, rural households with low income level benefit less from the increase in DPC. These findings emphasize the differentiated impact of DPC across demographic and socio-economic strata. Fourth, drawing on the Fogg Behavior Model, this paper constructs a theoretical framework for how DPC affects UFCP. The mechanism analysis indicates that household income and health awareness of the household head are essential for promoting UFCP in rural households.

## 7 Enlightenments

Drawing on these findings, we propose the following recommendations for developing countries seeking to harness digital payment technologies to enhance the well-being of rural residents: First, it is essential to advance the construction of rural digital infrastructure, including expanding internet access and mobile payment platforms. Enterprises should be encouraged to build diversified application scenarios for digital payment and innovate digital payment products and services. The government should strive to enhance the digital literacy of rural residents through targeted training programs, creating favorable conditions for the widespread adoption of digital payment. Second, the health education system and mechanisms for public opinion guidance should be improved. Schools should offer courses on food nutrition and health management. Targeting adolescents and middle-aged to elderly groups, a combination of traditional and new media should be used to disseminate knowledge on food nutrition and chronic diseases to rural residents, thereby enhancing their awareness of balanced diets. Third, we should fully unleash the income-increasing effect of digital payment capability. It is necessary to promote the training of rural residents in digital technology and their ability to use digital technology services for business and entrepreneurship. We should also improve the non-agricultural employment quality of rural residents, optimize the allocation of resources, and increase the income of rural households through multiple channels. Finally, developing the nutrition industry and online personalized health services plays an important role in the upgrading of the food consumption pattern in rural households. By embedding these strategies within a broader international framework, developing countries can leverage digital payment not only as a financial tool but also as a transformative mechanism for inclusive rural development and global nutritional improvement.

## 8 Limitations

Despite the valuable insights provided, this study has certain limitations that warrant acknowledgment. First, the cross-sectional nature of the 2020 CRRS constrains causal interpretation. While the data provide rich insights, they do not allow examination of how digital payment capacity and food consumption patterns evolve over time, including across different stages of the pandemic. Consequently, causal inferences about temporal dynamics should be interpreted with caution, and future panel or repeated cross-sectional data would be valuable to validate and extend our findings. Second, despite controlling for a range of observable confounders, endogeneity cannot be ruled out in a purely observational design. Potential sources include unobserved time-invariant factors, reverse causality, and measurement error in self-reported data, which may bias estimated effects. Third, unobserved cultural or regional factors may modulate the strength and direction of the estimated relationships. Future work leveraging experimental designs or quasi-experimental methods, as well as models that explicitly accommodate regional heterogeneity, would strengthen causal identification and enhance the generalizability of conclusions regarding the role of digital liquidity access in rural dietary transformation.

## Data Availability

The original contributions presented in the study are included in the article/supplementary material, further inquiries can be directed to the corresponding author.

## Footnotes

^1^ https://www.fao.org/china/news/detail/zh/c/1708195/

^2^Meat primarily includes pork, chicken, fish, beef, and mutton; eggs refer primarily to chicken eggs; milk includes primarily liquid dairy products and milk powder; quantities are measured in kilograms.

^3^ http://www.caict.ac.cn/kxyj/qwfb/bps/202007/P020200703318256637020.pdfw
